# Antisepsis

**Published:** 1887-11

**Authors:** M. H. Chappell


ARTICLE V.

### ANTISEPSIS.

BY M. H. CHAPPELL, D. D. S.

*Sepsis*—Putrefaction.

Anti "*Sepsis*"—To prevent, arrest, or destroy germs; chemical changes or putrefaction.

*Putrefaction*—The disorganization of animal tissue, or the formation of new organisms, microscopic or otherwise, relating to *corpuscle* life.

*Fermentation*—The action by chemical re-agents, as a result of light, heat, etc., and the development of corpuscle life, bioplasm in vegetable substances.

Antiseptic remedies are used to destroy, arrest or prevent, any of the conditions which would result in inflammation or terminate in suppuration or putrefaction.

The terms used, such as putrescence, disinfectants, escharotics, septicæmia, pyæmia, etc., must all be duly classified and used in their proper order.

Antiseptic treatment in wounds is to prevent that condition of the tissues, in its process of repair, either from the ravages of specific inflammation or from what we formerly called granulative inflammation, which did not throw off laudable pus, and to arrest the process of disorganization, putrescence, gangrene, dry or soft, or the formation of mephite gas, which is so easily detected by the smell.

Therefore, antisepsis treatment is the rational medication of wounds so as to prevent inflammation and restore the part by promoting the organization of lymph into formative or reparative tissue. This is done by the use of agents that will prevent the proliferation of micro-organisms, or if developed and manifesting their presence, resulting in fever of the parts, leading to inflammation and ulceration. Antiseptic remedies are of various degrees of strength—some are restrainers, others are destroyers, hence some germs may be developed, but rendered sterile by low strength preparatives, while the low strength in using are aimed as preventers or restrainers, and not destroyers. Many of these agents are pleasant, not powerful in action, but limited in their powers as restrainers, while others are non-offensive, but powerful in action, effective in purpose, and satisfactory in results.

The surgeon of to-day who honors his profession is an enthusiast in the science of antisepsis, or, rather, anti-germi-sepsis.

The germ theory in the treatment and management of wounds, as well as in disease and its prevention, is the king of theories in the healing art to-day.

Germicides are the antiseptics for us to consider, investigate, and make practical application thereof.

The surgeon who now takes a pride in his profession will not attempt to perform any surgical operation unless he has the means available that are essential to prevent germs coming in contact with the wound, either from his hands, instruments, atmospheres, flaculic or other means, and all these must be antiseptized.

We, as dental surgeons, have operations that result in very unpleasant terminations, and as the conditions for major and minor surgery are the same only in extent and responsibility, we must be informed and governed accordingly.

In the treatment of pulpless teeth, putrescent canals, ulcerating peri-dental membranes, alveolar abscesses, pyorrhœa alveolaris, in the treatment of carious cavities in teeth prior to filling, and wounds of the mouth and face, give us a large field for practice. There are but few, if any, mouths but that are daily offering in some parts conditions for the development and growth of germs, either formative or nomadic. These germs may be from an animal or corpuscle origin, owing to their cause or resting place, while others are vegetable, and either may produce similar results.

It is a known fact that vegetable substances can be anæsthetized and held dormant, as well as the highly organized corpuscle life. Hence, we see that some strengths of antiseptics act only as restrainers on germs that will regain life, and may be more definitely fertilized and become aggressive in diseased action.

Our pharmacists, and those who take a pride in developing new remedies in materia medica, have given us a long list of antiseptics and germicides. It is not safe to adopt *one idea* only in thought—mental philosophy—for it endangers the machinery for developing other thoughts; hence insanity, with its dangers and misfortvnes, even in the realm of thought. So it is if we adopt "one-idea" remedies. We must comprehend the whole, and observe that the world moves and the tides flows.

Time will not permit a full discussion of this subject, and I will present only a few remedies and their uses:

Bichloride of mercury has for ages been considered too violent a poison to use as a medicament, but as the lightnings are tamed and trained to do our service, so is corrosive sublimate. Its most valuable use is that of a germicide. It is pleasant in use and reliable in destroying germs and preventing inflammation, and assists in speedy recovery. I use it in various strengths, and it is claimed to be a restrainer, or even destroyer, at 1–60000.

For the purpose of practical use, the following table of weights of fluids will not be amiss:

1 gallon distilled water, 10 lbs.
1 pound, 5,760 grains.
1 ounce, 480 grains.
1 drachm, 60 grains.
1 minim, 1 grain.

Apothecaries' weight.

Hence the varied strength can be approximated. For irrigating purposes, such as washing out with syringe or textile siphon, the 1–1000 to 1–2000 strength is required.

For dental use I irrigate with syringe all mouths that are any way affected with putrescence. Warm water, or rather rain water that has recently been boiled or distilled, is required for the solutions in immediate use in cases heretofore described. And for all carious cavities wipe out with pledget of cotton saturated with the 1–200 solution; then dry with borated cotton and paris green and bichloride powder.

In putrescent canals irrigate with syringe and 1–1000 solution. Be observant that in exploring no debris is forced through the foramen, and if a discharge is flowing this must not be checked. Pack the cavity with borated cotton, wet with the bichloride solution. Fill mouth of cavity temporarily with oxyphosphate.

In pyorrhœa alveolaris cleanse by removing all extraneous substances, then apply by irrigating with the warm water solution 1–100.

The stage of disease and its character will in some cases indicate some remedy of different properties, like iodine or sulphuric acid, as hereinafter mentioned.

Some may inquire as to the toxicant dangers and in what would they consist—salivation, with griping pains, but no fatal results. In no case of dental surgery is there any danger of poisoning.

I can not pass this part of my subject without insisting on each of you to become missionaries among your family physicians and surgeons and inquire of them if they "have the light" of *antisepsis* in the treatment of wounds, and especially in the use of bichloride of mercury 1–1000 solution. If they *have not this light*, proceed to tell them of the "balm in Gilead," and of the wonderful results.

When you go home prepare your solutions, if you have not already done so, and make practical the information you have on the subject.

Carbolic acid possesses varied properties. Escharotic, germicide, germ restrainer, disinfectant, antiseptic, and stimulation, in accordance with the strength used. We have observed the application of carbolic acid, full strength, to putrescent canals and tresh wounded extirpated canals alike, when neither was indicated.

When we desire a line of demarcations in a cold abscess, and the curing, like bacon is made of the decalcified residum in white or yellow decay in dental caries; when we wish to fill over and destroy or restrain the germs therein, then full strength is required. But as a germ restrainer or killer, 1-30 solution will be sufficient, either as a disinfectant or antiseptic.

Peroxide of hydrogen is a wonderful effervescent agent when it comes in contact with pus, blood, or saliva. In cleansing or freeing abscesses, or putrescent pulps, or foul gums, of all puss, it is valuable. But owing to the trouble of losing the extra equivalent of oxygen, in some cases our success is not satisfactory, although a good article gives good results.

Iodine in tincture is not only a germicide, but it is the most powerful and successful *resolvent* stimulant known. It promotes the process of *resorption* of the waste tissue, and facilitates the lencocyte scavengers in the blood to seize and destroy any germs or broken down pabulum that can not be formed into protoplasm, and it is eliminated through the kidneys or other excretory glands. It promotes a flow of organizable lymph for provisional tissue. Hence the indications in all debilitated, sluggish, or specific troubles, where healing will not proceed, then iodine indicated, and the strength required is suggested by the prognosis. In full strength, or a syrup of the metal, iodine is a powerful escharotic, while a tincture, and even in solution, the resolvent stimulant qualities are valuable.

Sulphuric acid is a valuable agent in the mouth. If we use it full strength, it is an escharatic and powerful germicide. In scaling and polishing teeth, I use a solution after scaling and before polishing, to cleanse all the carious ends of enamel roots when the teeth are attacked with green stain—a form of caries—and if decalcification takes place, sulphate of lime is left which the green stain decay will not attack soon.

In children's teeth where the secretions of mucus are vitiated, caries, white, a valuable daily wash, is made 1–100 solution aromatic sul. acid, preparatory to further operations in removing carious debris and correcting the mucus glands.

Iodoform is an excellent dressing for putrescent canals, if not the best, when sealed and changed daily.

Benzoaic acid I use with borated cotton for packings and other dressings.

Sanitas, eucalyptus and other antiseptic agents are mild and pleasant germicides. We must admit that with many individuals some remedies have but little sanitive effects.

For two years I have been a student and an investigator into the practice of antisepsis, with the aid of standard works like Wythe on Surgery, Pilcher and Morris (1887 edition) on Wounds.

I have in my possession two photos of a little girl four years old living in Kennard, near our place, and its mother, having become violently insane, with three strokes of a hatchet crushed its skull to the depth of one and one-half inch, making a ghastly wound. The papers reported the killing of the child. As life was apparent, Dr. E. O. Holloway, of our place, who is an enthusiastic advocate of antisepsis, was called, and soon removed the broken bones and nearly a teacupful of brains, and then used nothing but the 1–2000 bichloride solution on borated cotton dressings. After eight weeks' treatment the child is now running about as well as usual, save slight paralysis and a severe scar. The mother is yet in the State hospital for the insane. This is the most wonderful case I have known in our neighborhood, and all to the credit of antisepsis.—*Transactions of the Indiana State Dental Association.*

